# Extending health messaging to the consumption experience: a focus group study exploring smokers’ perceptions of health warnings on cigarettes

**DOI:** 10.1080/16066359.2019.1653861

**Published:** 2019-08-29

**Authors:** Crawford Moodie, Rachel O’Donnell, Joy Fleming, Richard Purves, Jennifer McKell, Fiona Dobbie

**Affiliations:** a Institute for Social Marketing, School of Health Sciences and Sport, University of Stirling, Stirlingshire, Scotland;; b Usher Institute of Population Health, College of Medicine and Veterinary Medicine, University of Edinburgh, Stirlingshire, Scotland

**Keywords:** Tobacco, health warnings, public policy

## Abstract

**Introduction:** While most countries require health warnings on cigarette packs, the Scottish and Canadian Governments are considering requiring health warnings on cigarette sticks.

**Methods:** Twenty focus groups were conducted in Glasgow and Edinburgh (Scotland) with smokers (*n* = 120) segmented by age (16–17, 18–24, 25–35, 36–50, >50), gender and social grade, to explore perceptions of cigarettes displaying the warning ‘Smoking kills’ on the cigarette paper and any demographic differences in how smokers responded to these.

**Results:** A warning on each cigarette was thought to prolong the health message, as it would be visible when a cigarette was taken from a pack, lit, left in an ashtray, and with each draw, and make avoidant behavior more difficult. That it would be visible to others was perceived as off-putting for some. It was felt that a warning on each cigarette would create a negative image and be embarrassing. Within several female groups they were viewed as depressing, worrying and frightening, with it suggested that people would not feel good smoking cigarettes displaying a warning. Within every group there was mention of warnings on cigarettes potentially having an impact on themselves, others or both. Some, mostly younger groups, mentioned stubbing cigarettes out early, reducing consumption or quitting. The consensus was that they would be off-putting for young people, nonsmokers and those starting to smoke.

**Conclusions:** Including a warning on each cigarette stick is a viable policy option and one which would, for the first time, extend health messaging to the consumption experience.

## Introduction

The guiding principles of the Framework Convention on Tobacco Control state that consumers should be warned of the risks associated with tobacco consumption (World Health Organisation 2005). Tobacco packaging is one way to communicate these risks, and for many countries warnings have been required on cigarette packs for decades. Hiilamo et al. (2014) explain that warnings on cigarette packs have typically progressed from the vague and unobtrusive to the specific, prominent and graphic. Indeed, almost 120 countries now require pictorial health warnings on packs, and in over 100 of these countries they must cover at least 50% of the main display areas (Canadian Cancer Society 2018).

As large pictorial warnings on packs become the global norm, what does this mean for the evolution of health messaging? One option that has recently captured the attention of policy makers is a warning on each cigarette stick (Hassan and Shui 2015; Moodie et al. 2015). In June 2018 the Scottish Government’s five-year tobacco control action plan included an action point to make cigarettes less attractive, either through the use of an unattractive color (Hoek and Robertson 2015) or the inclusion of a warning (Scottish Government 2018), and in October 2018 the Canadian Government launched a consultation on labeling for tobacco products, including the possibility of warnings on cigarette sticks (Health Canada 2018). Tobacco companies responding to the consultation opposed warnings on cigarette sticks, arguing that smokers are aware of the health risks and that Health Canada failed to provide any evidence that warnings would impact the decisions of people to start, continue or stop smoking (Nuthall 2019).

In this study, we focus on the response of smokers in Scotland to the warning ‘Smoking kills’ on the cigarette. Three quantitative studies in the United Kingdom (UK) have explored perceptions of cigarettes with this warning. The first was an in-home survey with 11-16 year olds (*n* = 1205) in 2014, with participants shown an image of a cigarette with this warning and asked about the impact on initiation, cessation and support. Almost three-quarters (71%) indicated that warnings on cigarettes would put people off starting to smoke, 53% thought that they would make people want to give up smoking, and 85% supported a warning on every cigarette (Moodie et al. 2017). In two online surveys, with 16-24 year old smokers and nonsmokers (*n* = 997) in 2015 (Moodie et al. 2019), and 16-34 year old smokers (*n* = 1766) in 2016 (Moodie, Hoek et al. 2018), participants were shown an image of a regular cigarette, cigarette with warning, and unattractively colored (green) cigarette, and asked to rate each on appeal, harm, and likely trial. Compared with the regular cigarette, smokers and nonsmokers rated the cigarette with warning and the green cigarette as less appealing and more harmful, and indicated that they would be less likely to try them (Moodie, Hiscock et al. 2018; Moodie et al. 2019). Two further studies have explored perceptions of regular (white cigarette paper with white or imitation cork filter) and dissuasive sticks (a cigarette with ‘Smoking kills’ on the cigarette paper, a cigarette displaying ‘minutes of life lost’ for each cigarette on the cigarette paper, a yellow cigarette, and a green cigarette) (Hoek et al. 2016; Lund and Scheffels 2018). Hoek et al (2016) conducted an online survey in 2014 in New Zealand with smokers (*n* = 313), using a Best–Worst Choice experiment and rating task, and found that each dissuasive cigarette was rated as less appealing than the regular cigarettes. An online survey with 16-20 year olds (*n* = 280) in 2016 in Norway found that the four dissuasive sticks were perceived as less appealing, worse tasting, more harmful, and less likely to encourage product trial, than the regular cigarettes (Lund and Scheffels 2018).

Two qualitative studies have explored the potential impact of including ‘Smoking kills’ on cigarettes. The first, in 2012, was focus groups with 16-24 year old female smokers (*n* = 49) shown four cigarettes with ‘Smoking kills’ displayed on the filter, horizontally on the cigarette paper, and either once or twice vertically on the cigarette paper (Moodie et al. 2015). The cigarette displayed vertically on both sides of the cigarette paper was considered most salient. Having a warning on cigarettes was viewed as unappealing for some participants, a reminder of the associated risks, and off-putting primarily because of the perceived discomfort of being observed by others smoking a cigarette displaying this message (Moodie et al. 2015). The second study, in 2014, involved interviews with marketing experts (*n* = 12). A warning on cigarettes was suggested to confront smokers, deter nonsmokers, signal to youth that it is not cool or clever to smoke, prolong the health message, serve as a continual reminder of the associated risks, and undermine the use of an alternative carrier for those decanting cigarettes to avoid exposure to on-pack warnings or plain packaging (Moodie 2016).

Given the lack of research on a policy option that is being considered in more than one country, we extend the only qualitative study to have explored smokers’ perceptions of warnings on cigarettes with a more diverse sample.

## Methods

### Design and sample

Twenty focus groups were conducted between January-March 2015 with 120 smokers in the two most populated cities in Scotland (Glasgow, Edinburgh) to explore their perceptions of cigarette packaging, pack inserts promoting cessation (Moodie 2018b), and also cigarette design, which is the focus of this paper. Focus groups were employed as they are helpful for gaining insight into how a particular sample may respond to a novel concept, in this case how smokers respond to cigarettes with the warning ‘Smoking kills’. Groups were segmented by gender, age (16–17, 18–24, 25–35, 36–50, 51+), and social grade (ABC1, C2DE) to allow us to explore any demographic differences, see Table 1. Social grade was determined by the occupation of the main income earner within the household using the National Readership Survey, an established classification system in the UK with grades A, B and C1 signifying middle class groups and C2, D and E working class groups (National Readership Survey, undated) Table 1.

**Table 1.** Demographics of, and number within, each group.

Group number	Age group	Gender	Social grade	Number in group
1	16–17	Female	ABC1	7
2	16–17	Female	C2DE	7
3	16–17	Male	ABC1	5
4	16–17	Male	C2DE	6
5	18–24	Female	ABC1	5
6	18–24	Female	C2DE	7
7	18–24	Male	ABC1	7
8	18–24	Male	C2DE	5
9	25–35	Female	ABC1	6
10	25–35	Female	C2DE	6
11	25–35	Male	ABC1	5
12	25–35	Male	C2DE	6
13	36–50	Female	ABC1	5
14	36–50	Female	C2DE	6
15	36–50	Male	ABC1	6
16	36–50	Male	C2DE	5
17	>50	Female	ABC1	6
18	>50	Female	C2DE	7
19	>50	Male	ABC1	7
20	>50	Male	C2DE	6

Participants were recruited in Glasgow or Edinburgh by market researchers using convenience sampling. Market researchers were asked to intercept potential participants in the street and explain that the study was concerned with perceptions of tobacco packaging and warnings. Demographic information (age, gender, social grade) and smoking behavior (smoking status, smoking frequency, consumption) was captured by a recruitment questionnaire. The inclusion criteria were that participants were within one of the gender, age and social grade groups and smoked cigarettes at least once a week. Groups (14 in Glasgow, 6 in Edinburgh) took place in a venue suitable for hosting a group discussion (e.g. hotel function room) and convenient for participants.

### Procedure

Market researchers provided eligible participants with an information sheet, which detailed the study. Those interested in participating were asked to complete a consent form. Both the information sheet and consent form explained that the study was anonymous and that participants could withdraw at any time and did not have to respond to any questions. In addition to reminding participants of confidentiality, anonymity, their right to withdraw and not to respond to questions at the start of each group, they were informed that their views may differ and when answering they should not be influenced by anyone else within the group or the interviewer(s). Groups typically lasted 90 minutes, after which participants were debriefed about the study and received an incentive fee (£25). Ethical approval was obtained from the Ethics Committee of the School of Management at the University of Stirling.

**Figure 1.**
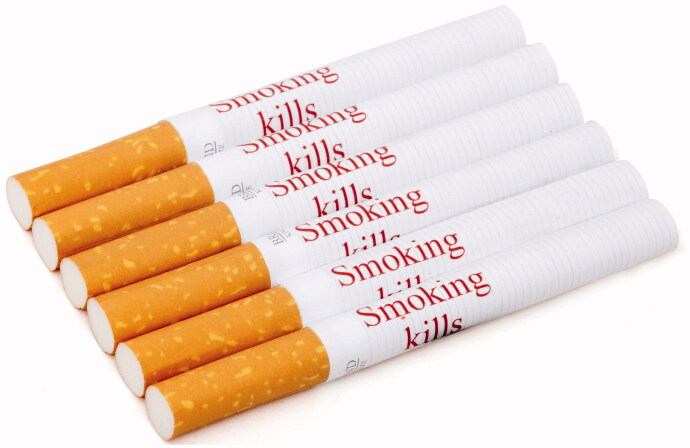
Warning on cigarettes.

Groups were moderated by one or more of the research team (CM, RP, JM, FD), experienced facilitators working at the University of Stirling. Only the interviewer(s) and participants, who were not known to the interviewer(s), were present during the groups. A semi-structured topic guide ensured that groups were asked a common set of questions, but otherwise participants lead the discussion, with the interviewer(s) following up on comments made. Focus groups allow participants the opportunity to interact with stimuli, which is of particular value when the stimuli are novel, and within each group participants were shown cigarettes displaying the warning ‘Smoking kills’ twice on the cigarette paper (see Figure 1) and given these to hold. They were initially asked to ‘Imagine the warning Smoking kills was displayed like this on every cigarette, irrespective of brand’ and given time to discuss this. All groups were subsequently asked about how warnings on cigarettes would make them feel, what image they created, awareness of harms, and impact on their own and others behavior, see Supplementary file.

### Analysis

A thematic analysis was employed. The transcripts were initially checked against the audio files to ensure accuracy and then reviewed using an iterative approach, with content categorized by two members of the research team (CM, JF) according to inductively developed thematic codes. Seven main themes were identified (Salience, Avoidance, Appeal and image, Perceived response from others, Emotional response, Impact on smoking and cessation-related behaviors, Impact on young people and nonsmokers). Two different researchers (RO, RP) then reviewed each transcript to identify quotes that may have been missed and that could meaningfully add to the main themes. In the Results, quotes are followed by details of the age group, gender (M or F), and social grade (e.g. 16–17 F, ABC1), and where there are demographic differences these are mentioned.

## Results

### Salience

The warning ‘Smoking kills’ on cigarette sticks was considered prominent, and difficult to miss, e.g. ‘*It’s right in your face*’ (36–50 F, C2DE). It was thought to prolong the health message and be a constant reminder, given that it would be visible while taking a cigarette from a pack, lighting it, with every draw, and when it is in the ashtray.

As soon as you open your packet that is what you are going to see, just “Smoking kills” on every fag that’s there, and then you are going to see it every time you take, like, when you are taking a draw (16–17M, C2DE)

I think there is something more memorable about that. When you are lighting (it) you have to look at the cigarette (36–50M, ABC1)

You are going to see it fifteen, twenty times a day (25–35M, ABC1)

In all age groups there was mention of warning wear-out, e.g. ‘*After time, you might become desensitized to it because you see it all the time*’ (18–24 F, ABC1).

### Avoidance

As warnings would be visible throughout the smoking experience, from taking a cigarette out of a pack to smoking it and even discarding it, at which point the message may become visible to others, it was generally thought to make avoidant behavior more difficult.

When you’re smoking it you can’t really hide, you can hide the pack but you can’t hide the cigarette (16–17F, C2DE)

It’s putting a point across a bit more. You know, the packet can go back into your jacket, whereas you’ve got the cigarette in your hand, it’s going to be there for everybody to see (25–35M, C2DE)

In an attempt to avoid the warning, in three female groups comments were made about using a pen to conceal it, e.g. ‘*I’d be looking for a permanent marker, just to draw over it*’ (25–35 F, C2DE). Within several, mostly younger male groups, it was suggested that they would switch to rolling tobacco, e.g. ‘*You’d just buy roll ups*’ (16–17 M, ABC1), or use rolling tobacco papers (known as ‘skins’) to cover the warnings, e.g. ‘*I’d just stick a skin round it*’ (25–35 M, C2DE). However, when the moderator mentioned that rolling tobacco papers would also display a warning, some participants suggested that they would consider quitting.

I would actually rather sprinkle the tobacco inside a book and then put it into a skin, a normal skin

**What if the skin has got it on it as well? (Moderator)**

What like just a normal skin?

**Every cigarette paper has got the message on it as well? (Moderator)**

I’d stop smoking then (18–24M, C2DE)

### Appeal and image

Within some, mostly younger female groups, cigarettes with warnings were considered horrible and the antithesis of fashion, e.g. ‘*That is in no way cool or trendy*’ (18–24 F, ABC1).

That really looks crap and that’s great, and it should be looking crap (18–24F, C2DE)

That is certainly not a glamorous fag (36–50F, ABC1)

The importance of image was raised, e.g. ‘*It’s all about image*’ (18–24 F, C2DE), with cigarettes with warnings creating a negative image, particularly within female groups. It was felt that for those smoking a cigarette with a warning it would make them appear rather foolish, and associations were also made with older people, and long-term or addicted smokers.

It’s maybe not as cool, ken (know), it’s a bit like having ‘Idiot Stick’ in your hand cause you’re standing with that (25–35M, C2DE)

Someone who had been smoking for a long time and couldn’t give up. Didn’t have the willpower – older (18–24F, ABC1)

Someone who is totally addicted and doesn’t really care (50 + F, ABC1)

### Perceived response from others

As warnings on cigarette sticks would be visible to other people, e.g. ‘*It’s hard hitting and people would see it*’ (36–50 M, ABC1), within several groups it was suggested that they may be perceived negatively, or questioned or judged, by others, particularly nonsmokers or their children.

If you were a non-smoker and you were standing talking to somebody – maybe one of the boys smoking – you’re standing, you’re a non-smoker and you seen something that says ‘Smoking kills’ on it, you’d maybe think ‘That guy is, he’s a bit mental’ (36-50M, C2DE)

The main problem for me would be people being able to see. They’d question you more on it, like, ‘Why are you doing that?’ (16–17F, ABC1)

The message that that carries when you’re standing smoking that, to other people. If I’m sitting there smoking that and you look at that, you’re going, ‘Why is she smoking that when it says, clearly says Smoking kills’? (36–50F, C2DE)

Some participants, mostly younger females, thought that they or others would be less likely to want to smoke in front of others.

I wouldn’t want to smoke it in front of people (16–17F, C2DE)

It’d stop people from wanting to smoke because people around you would see it as well (16–17F, ABC1)

I think there would be a lot less public smoking (18–24F, ABC1)

### Emotional response

Participants made a number of comments about how they would feel holding and smoking cigarettes with warnings, with several groups, including all 16-17 year old groups, mentioning embarrassment, e.g. ‘*That might be something that would work with me, that might turn me off it. I think I would be very embarrassed to hold that*’ (50 + M, C2DE). Some participants said that they would only feel embarrassed when in the company of nonsmokers.

Just with non-smokers it would be embarrassing (16–17F, ABC1)

It’s not so bad between people who smoke cause you are in the same position, but if you were with friends who don’t smoke, it would be a bit embarrassing (16–17F, C2DE)

Within several female groups warnings on cigarettes were described as depressing, worrying and frightening, e.g. ‘*That would scare me*’ (25–35 F, ABC1). Some participants also thought that they would feel more negatively about smoking these cigarettes.

**So how would you feel holding this? (Moderator)**

Dreadful

You wouldn’t feel good at all

It’s horrible (50 + F, ABC1)

Maybe gives you a wee bit of a guilt trip as well cause you’re standing there with that in your hand and you’ve got kids to think about (25–35M, C2DE)

Several participants said that these negative feelings would be less of a concern when in the company of other smokers, e.g. ‘*You wouldn’t feel as bad cause everybody would have it on their cigarettes*’ (16–17 F, ABC1).

### Impact on smoking and cessation-related behaviors

Within every group there was mention of warnings on cigarettes potentially having an impact on other people, themselves, or both. Participants, particularly within female higher social grade groups, felt that they would be off-putting for everyone.

I can’t imagine anyone smoking that (16–17F, ABC1)

I think that would be good to stop people. On the cigarette, actually on the cigarette (25–35F, ABC1)

Some participants suggested that it would make them question their smoking behavior, e.g. ‘*When your cigarette is burning down you would be seeing it, I would think twice before smoking a cigarette*’ (50 F+, ABC1), while others, mostly males, felt that they would stub cigarettes out early or reduce consumption.

A lot of people wouldn’t even smoke it, and you wouldn’t even get a couple of draws

Aye because you would throw it away before it got to the end (16–17M, ABC1)

Believe it or not, no, I think it would make me stub it down (50F+, C2DE)

I don’t think I’d smoke fags that much (18–24M, C2DE)

A number of comments were made about warnings on cigarettes helping them, and other smokers, to quit, e.g. ‘*It can, like, sort of reinforce them, like…‘I actually have reasons to quit and I’m just being reminded every single time that I smoke*’ (18–24 M, ABC1), with some participants stating that they would not smoke or buy them.

I think it would make me want to stop (50 + F, C2DE)

The cigarette that gives you the warning on it, that actual cigarette is a total ‘no’ for me. That would definitely put me off (36–50F, C2DE)

If I am honest that would probably put me off

It doesn’t look so appealing

I wouldn’t smoke that like

I actually wouldn’t buy fags (18–24 M, C2DE)

Participants within several, predominantly male and lower social grade groups, stated that the inclusion of warnings on cigarettes would not alter their smoking behavior as they have already purchased the pack, are aware of the health risks, lack the willpower to quit, are addicted, and as they consider health problems or financial reasons the main drivers of quitting.

If you want to smoke, you’ll smoke

Warnings are nae (not) going to make any difference to an ardent smoker (25–35M, C2DE)

It’s an addiction (36–50M, C2DE)

I’m 62 and no amount of warnings is going to stop me smoking. To me I’ve already done the damage (50 + M, C2DE)

### Impact on young people and nonsmokers

Myriad comments were made about the potential impact on young people and nonsmokers. A warning on the cigarette stick was thought to be an effective way to communicate a health message to those not exposed to cigarette packs.

When you were, like, 13 and you were walking to school and one of your mates comes out and says, ‘Oh, I stole three cigarettes’, you know, like, pass it to someone that’s never smoked before in their life, never seen a packet, and they’re about to light up and it’s, like, ‘Smoking kills’ on the side of it, that might have an effect then (18–24M, ABC1)

The general view was that warnings on cigarettes would be a deterrent for some young people, nonsmokers and those just starting to smoke, e.g. ‘*Kids are going to look at it and they will be put off it*’ (25–35 M, C2DE).

Some people will stop smoking, but it’s more the people that dinnae (didn’t) smoke you want to, like, totally discourage them fae (from) even starting and I think that’s, like, effective that way (25–35M, C2DE)

It’d make you think twice about it when you were younger (18–24F, C2DE)

I think for new smokers looking at it, they’d be like ‘oh I don’t want to do that’ (16–17F, C2DE)

Female participants in particular reflected on whether they would have started smoking if they had seen, or only had access to, cigarettes displaying warnings. Some thought that they would not make a difference as other factors, such as peer pressure, were more important, whereas others felt that it may have stopped them.

I don’t think I would have started if I saw my friends smoking that (16–17F, C2DE)

If you were going into your mum’s fag packet and you were bringing something out and it was saying ‘smoking kills’ and that… you’d maybe be like, oh I better put that back (18–24F, C2DE)

I think it would have prevented me from starting because… it was cool, you know, to be doing it, my big sister did it, my friends did it, but if there was a blatant message on the cigarettes, I think at 14 I was intelligent enough to go no way, I mean I knew it was bad but I didn’t know it was as bad (25–35F, ABC1)

## Discussion

It is argued that health warnings should be the first thing that smokers see before buying their cigarettes and the last thing they see before lighting up (Kaiserman 1993). However, smokers are not necessarily exposed to warnings when they take a cigarette, including in markets with plain packaging, one of the core aims of which is to prevent pack design from detracting from the warnings. In Australia, for instance, there is evidence that some smokers use cases, re-use fully-branded packs, or cover or conceal packs (Wakefield et al. 2015; Hardcastle et al. 2016; Yong et al. 2016), and eye-tracking research has shown regular smokers to fixate on the branding rather than the warning on plain packs (Maynard et al. 2014). The general view in this study was that warnings on each cigarette stick would make avoidant behavior more difficult as smokers typically see a cigarette when taking it from a pack, lighting it, leaving it in an ashtray, and smoking it. What warnings on cigarettes would do, for the first time, is extend the health message to the actual consumption experience (Moodie 2018a).

Warnings on cigarettes would also be visible to others, which some participants thought may lead to them being perceived negatively, questioned or judged. Younger females, in particular, suggested that they would be less inclined to smoke in company, consistent with past research which found that some young women smokers were put off by the perceived discomfort of being observed by others smoking a cigarette displaying this message (Moodie et al. 2015). Just as the first five countries to have implemented plain packaging predict that this policy will lead to reduced exposure to secondhand smoke (Moodie et al. 2019) given participants’ concerns about smoking cigarettes displaying warnings in front of others this would appear a likely outcome with respect to this measure, particularly as the cigarette stick, unlike the pack, cannot be concealed. As almost 900,000 nonsmokers are estimated to die from secondhand smoke each year (World Health Organisation 2019), any policy options that may help to reduce this toll merit consideration.

The product experience includes its perception, cognitive associations, the emotions it elicits, and evaluative judgments (Schifferstein and Hekkert 2008). Our findings suggest that cigarettes with warnings would, at least for some people, create a very negative product experience. They were perceived to be horrible, associated with long-term or addicted smokers, and considered embarrassing, depressing and frightening, particularly among younger females. While some participants suggested that they would only experience negative thoughts and feelings in the company of nonsmokers, others thought that they would reduce the enjoyment of the smoking experience. In every group it was thought that warnings on cigarettes could change certain smoking behaviors (e.g. stubbing cigarettes out early or reducing consumption), encourage or lead to quitting, or deter young people, nonsmokers and those starting to smoke. This adds to online surveys which found that the warning ‘Smoking kills’ on cigarettes, in comparison to regular cigarettes, were associated with a lower likelihood of perceived trial (Lund and Scheffels 2018; Moodie, Hiscock et al. 2018; Moodie et al. 2019), and an in-home survey in which more than half of participants thought that having this warning on cigarettes would encourage cessation and almost three-quarters that it would discourage initiation (Moodie et al. 2017). These studies, collectively, suggest that warnings on cigarettes may have the potential to impact the decisions of people to start, continue or stop smoking, contrary to tobacco companies claims that there is no evidence to suggest that this would be the case (Nuthall 2019).

In terms of limitations, as this would have been the first time that the sample had seen warnings on cigarettes, their novelty may have influenced how they responded. This would of course be the case for all smokers in any country that required a warning on each cigarette, but nevertheless this study provides no insight into how smokers would respond to warnings on cigarettes over time. It is also possible that the findings were influenced by socially desirable responding. As wear-out is an issue with all warnings (Fischer et al. 1993), with habituation raised within the groups, future research should consider additional, appropriate warning messages (Drovandi et al. 2018; Drovandi et al. 2019a, 2019b, 2019c). Given that some participants suggested that they would switch to rolling tobacco or use rolling tobacco papers to cover warnings on cigarettes, research with smokers of rolling tobacco would be fruitful, particularly in markets where this product has a significant share of the tobacco market.

It is suggested that all marketing tools should be used to promote health (Kaiserman 1993). As the cigarette is an increasingly important form of marketing for tobacco companies (Smith et al. 2017; Moodie, Thrasher et al. 2018), particularly as large on-pack health warnings and plain packaging have reduced the promotional power of the packaging, then it is clearly a suitable platform for delivering health messaging. It would also be appropriate to do so given that cigarettes, which are responsible for most tobacco related mortality and morbidity, continue to dominate the global tobacco market and are predicted to remain the most popular form of nicotine consumption by 2050, even with the continued growth of ‘next generation’ nicotine products (Hedley 2015).

## Supplementary Material

Supplemental_Material
