# Supplementary material for: Extending health messaging to the consumption experience: a focus group study exploring smokers’ perceptions of health warnings on cigarettes
Source: Addict Res Theory. 2019 Aug 29;28(4):328–34. doi: 10.1080/16066359.2019.1653861 (PMC7454525; doi:10.1080/16066359.2019.1653861)
Supplement: Supplemental_Material [file IART_A_1653861_SM2227.docx]

**Core questions used in topic guide**

Imagine the warning Smoking kills was displayed like this on every cigarette, irrespective

of brand.

Think about the smoking experience, taking a cigarette from a pack, lighting it, smoking it,

would it change anything?

So how would you feel holding this?

What image would you associate with someone you saw smoking this?

Do you think that would make you more aware of the dangers?

Do you think it could have an impact on how you think about smoking?

Do you think this may have an impact on others?
